# Presbycusis: do we have a third ear?^☆^

**DOI:** 10.1016/j.bjorl.2015.12.006

**Published:** 2016-03-29

**Authors:** Luis Roque Reis, Pedro Escada

**Affiliations:** Faculdade de Ciências Médicas, NOVA Medical School, Centro Hospitalar de Lisboa Ocidental (CHLO), Hospital Egas Moniz, Departamento de Otorrinolaringologia, Lisbon, Portugal

**Keywords:** Presbycusis, Sensorineural hearing loss, Speechreading, Presbiacusia, Perda auditiva neurossensorial, Leitura labial

## Abstract

**Introduction:**

Age-related hearing changes are the most frequent cause of sensorineural hearing loss in adults. In the literature no studies exist concerning the importance of speechreading in individuals with presbycusis. Equally, no such studies have been carried out with speakers of the Portuguese (Portugal) language.

**Objectives:**

To evaluate whether the intelligibility of words in presbycusis is improved by speechreading, in such a way that looking at the interlocutor's face while he is talking functions like a “third ear”, and to determine the statistical relevance of the intelligibility improvement by speechreading.

**Methods:**

Eleven individuals (22 ears) with bilateral and symmetrical sensorineural hearing loss compatible with presbycusis were evaluated. The subjects were aged between 57 and 82 years, with an average of 70 ± 11.51 years and median of 69.5 years. A complete medical and audiological profile of each patient was created and all patients were submitted to a vocal audiogram, without and with observation of the audiologist's face. A descriptive and analytical statistical analysis was performed (Shapiro–Wilk and *t* pairs tests) adopting the significance level of 0.05 (5%).

**Results:**

We noticed better performance in intelligibility with speechreading. The *p*-value was zero (*p* < 0.05), so we rejected the null hypothesis, showing that there was statistically significant difference with speechreading; the same conclusion was obtained by analysis of the confidence intervals.

**Conclusions:**

Individuals with presbycusis in this study, performed better on spoken word intelligibility when the hearing of those words was associated with speechreading. This phenomenon helps in such a way that observation of the interlocutor's face works like a “third ear”.

## Resumo

**Introdução:**

As alterações auditivas relacionadas com a idade são a causa mais frequente de perda auditiva neurossensorial em adultos. Não há estudos na literatura sobre a importância da leitura orofacial em indivíduos com presbiacusia e, considerando as particularidades de cada língua, que abordem esse tema em falantes de português de Portugal.

**Objetivo:**

Avaliar se a inteligibilidade das palavras na presbiacusia é melhorada pela leitura orofacial, de tal forma que olhar para o rosto do interlocutor enquanto ele está falando funcione como uma “terceira orelha”. Determinar a relevância estatística da melhora na inteligibilidade pela leitura orofacial.

**Método:**

Ao todo, 11 indivíduos (22 orelhas) com perda auditiva neurossensorial bilateral e simétrica compatível com presbiacusia, idades entre 57 e 82 anos (média de 70 ± 11,51 anos e mediana de 69,5 anos) foram avaliados. Um perfil médico e audiológico completo de cada paciente foi realizado, e todos foram submetidos a um audiograma vocal, sem e com a visualização do rosto do fonoaudiólogo. Uma análise estatística descritiva e analítica foi realizada (teste de Shapiro–Wilk e teste *t* pareado), adotando o nível de significância de 0,05 (5%).

**Resultados:**

Verificámos melhor desempenho na inteligibilidade com a leitura orofacial. O valor-*p* foi zero (*p* < 0,05), pelo que rejeitámos a hipótese nula, indicando que houve diferença estatisticamente significativa com a leitura orofacial. A mesma conclusão foi obtida por análise dos intervalos de confiança.

**Conclusões:**

Os indivíduos com presbiacusia tiveram melhor desempenho na inteligibilidade das palavras faladas quando a audição foi associada à visualização do rosto do interlocutor. Essa parceria auxilia de tal maneira que parece funcionar como uma “terceira orelha”.

## Introduction

Age-related hearing changes are the most frequent cause of sensorineural hearing loss in adults.^1^ Presbycusis is a gradual bilateral hearing loss associated with aging that is due to progressive degeneration of cochlear structures and central auditory pathways. This hearing loss usually begins at high frequencies and then progresses to sounds of middle and low frequencies.^2^ The age of onset and its evolution are related to interindividual variability, with genetics and environmental factors involved.^3^

Auditory difficulties faced by individuals with presbycusis can be compensated by using speechreading as a strategy, with the objective of assisting the recognition of the spoken message, and providing more effective communication.^4^ It is a process in which an observer comprehends speech by watching the movements of the speaker's lips, without hearing the speaker's voice.^5^ This speech recognition through visual cues includes not only the articulatory movement during speech, but also a careful observation of the speaker and their associated behaviors such as intonation, facial expression and body movements.^6^ In this study we chose the term “speechreading”, however, in the literature the terms lip reading and orofacial reading are also used.^7^

All individuals use speechreading. In fact, even individuals with normal vision and hearing use speechreading unconsciously and its use enables an increase of intelligibility in noise. Studies show that speechreading activates the auditory cortex in individuals with normal hearing in the absence of auditory stimulation.^8^, ^9^

There are no studies in the literature that evaluate the importance of speechreading in individuals with presbycusis. In addition, and considering that each language has its particularities, we note that no such studies have been carried out with speakers of the Portuguese (Portugal) language (PPt).

The authors hypothesize that in presbycusis the intelligibility of words is aided and complemented by speechreading, in such a way that observation of the interlocutor's face articulating the words functions like a third ear. This study aims to evaluate how speechreading increases the intelligibility in presbycusis and determine the statistical significance of improvement.

## Methods

This study was analyzed and approved by the Health Ethics Committee (CES) of *Centro Hospitalar de Lisboa Ocidental* (CHLO), Lisbon, on 16/03/2015. Individuals agreed to participate in the research and signed the informed consent.

### Participants

The sample included patients of the Otolaryngology Department of Egas Moniz Hospital in CHLO, that had been sent to the Audiology Department for audiological exams. It is an analytical and cross-sectional study, in which we used a convenience sample composed of 11 individuals (22 ears) who fulfilled the following inclusion criteria: aged 55 or more years, bilateral and symmetrical sensorineural hearing loss compatible with presbycusis, type A or As tympanogram (Jerger classification), vocal audiogram with Speech Reception Threshold (SRT) ≥ 40 dB, oral communication ability, PPt as first language and informed consent acceptance, after clarification of the procedures involved. Individuals with the following criteria were excluded: presence of tinnitus that could interfere with the audiometry, external or middle ear pathology, neurological and/or psychiatric disorders that could interfere with language, serious visual changes or no use of corrective lenses during the evaluation.

### Research tools

All patients were submitted to an evaluation protocol with a complete medical and audiological profile. An audiologic study (immittance, tonal and vocal audiograms) was performed, after which patients were reassessed in the office. If inclusion criteria were fulfilled, two new vocal audiograms would follow, after several weeks, sequentially without and with observation of the audiologist's face, in order to quantify the improvement of SRT with associated speechreading. All vocal audiograms without and with speechreading were performed by the same audiologist (female announcer) and the procedure was carried out with the knowledge of the patient. The examinations were conducted in a soundproof test room according to ISO 8253 and 389, with a Madsen Electronics audiometer, model Orbiter and 922 TDH39 earphones, noise-excluding headset ME70 and bone conductor B-71. On vocal audiometry the stimulus consisted of disyllabic phonemes with phonetic balance for PPt; the phonemes sequence was used randomly, with analysis of the following parameters: detection, reception and maximum discrimination of speech thresholds. The results were presented in the form of *x*–*y* graph (intelligibility curve), comparing the intensity of the stimulus with the percentage of words understood. Gender as a variable was not studied.

### Statistical procedures

The data were collected into a database and the statistical study was performed using the Statistical Package for the Social Sciences (SPSS), version 20.0 for Windows. In a first phase, we tested the conditions for application of statistical tests (normality and homoscedasticity), after which we were able to choose parametric or nonparametric tests. To evaluate the effect of speechreading on speech discrimination, we planned to use, in the case of parametric tests, the Student's *t*-test for paired samples, and in the case of nonparametric, the Wilcoxon test. We applied a significance level of 0.05 (5%) with 95% interval. We tested if there was a statistically significant difference between the SRT and the threshold of discrimination.

## Results

The age of the analyzed participants ranged from 57 to 82 years, with a mean age of 70 ± 11.51 years and a median of 69.5 years. SRT values were recorded with and without speechreading. This led us to two samples with quantitative and paired data. In other words, it is the same person before and after (respectively without and with speechreading). The main data referring to the characterization of the studied group is presented in Table 1. From the descriptive analysis, the result of the difference is on average 23.3 dB, with a median of 25 dB, a standard deviation of 7.9 and minimum and maximum values of 10 dB and 35 dB, respectively.

**Table 1:** Descriptive analysis of the results (SRT values, with and without SR: speechreading).

	Statistic	Std. error
**Without SR**		
*Mean*	57.22	5.34
*95% confidence interval for mean*		
Lower bound	44.90	
Upper bound	69.54	
*Median*	45.00	
*Variance*	256.94	
*Std. deviation*	16.03	
*Minimum*	40.00	
*Maximum*	80.00	

**With SR**		
*Mean*	33.89	4.39
*95% confidence interval for mean*		
Lower bound	23.76	
Upper bound	44.02	
*Median*	35.00	
*Variance*	173.61	
*Std. deviation*	13.18	
*Minimum*	15.00	
*Maximum*	50.00	

**Difference**		
*Mean*	23.33	2.64
*95% confidence interval for mean*		
Lower bound	17.26	
Upper bound	29.41	
*Median*	25.00	
*Variance*	62.50	
*Std. deviation*	7.91	
*Minimum*	10.00	
*Maximum*	35.00	

Comparing the average of the results, individuals showed better results with speechreading. There was a positive correlation between the improvement of the SRT and speechreading, with an average reduction of 23.3 dB. In order to apply the paired *t*-test, data from the difference (between the two samples) had to present a normal distribution (applicability condition). Therefore since the sample size was less than 50, we used the Shapiro–Wilk test (Table 2) to check for the sample normality.

**Table 2:** Results of testing the sample normality.

	Kolmogorov–Smirnov			Shapiro–Wilk		
	Statistic	df	Sig.	Statistic	df	Sig.
Without SR	0.333	9	0.005	0.802	9	0.022
With SR	0.194	9	0.200	0.919	9	0.382
Difference	0.139	9	0.200	0.971	9	0.906

**Figure 1:**
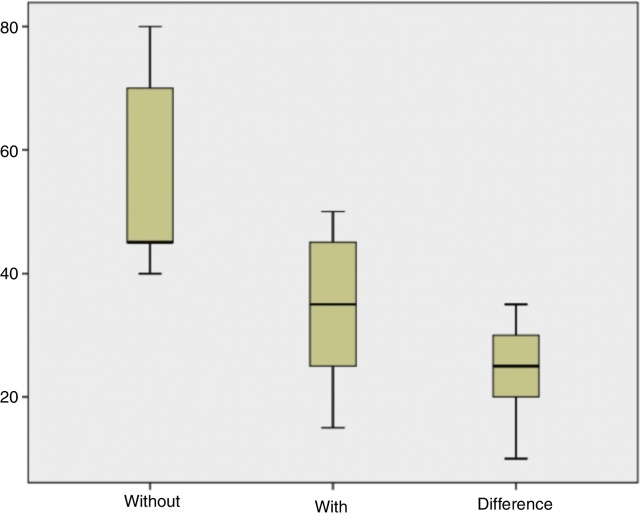
Box plots of data that display the variation in the sample.

The *p*-value in the Shapiro–Wilk test was 0.906 (greater than 0.05), so the null hypothesis was not rejected. Therefore we concluded that data of the difference had a normal distribution and the paired *t*-test could be used. Equal conclusion was drawn from the box plots analysis (Fig. 1). We established a null hypothesis for *t*-test-pairs *μ*_0_ equal to 0, that is, there was no difference between with and without speechreading.

As the *p*-value in the paired *t*-test (Table 3) was zero (<0.05), the null hypothesis was rejected and *H*_1_ accepted_._ Therefore, there was a statistically significant difference in discrimination between with and without speechreading. A similar conclusion was drawn from the confidence interval analysis (the non inclusion of zero is equivalent to say that RH_0._).

**Table 3:** Verification of the significance of the results using paired *t*-test.

Pair	Paired differences					*t*	df	Sig. (2-tailed)
	Mean	Std. deviation	Std. error mean	95% CI of the difference				
				Lower	Upper			
Without–with	23.33	7.91	2.64	17.26	29.41	8.85	8	0.000

## Discussion

The production of each phoneme triggers a characteristic position of the facial structures, such that someone with knowledge of the language can deduce, up to a certain extent, which phoneme was produced. The visual information of the speech articulation increases the auditory processing, when associated with direct information about the signal content, increasing discrimination of phonemes.

You can check this phenomenon in your daily clinical practice, when you speak to individuals who have hearing disabilities from presbycusis. If you speak initially with your mouth covered and then uncovered, at intensity near the patient's discrimination level, the observation of your face enhances intelligibility.

In the literature, there are no studies that address the importance of speechreading in intelligibility, in patients with hearing loss due to presbycusis and particularly in Portuguese language (PPt) speakers. This study demonstrates that these individuals demonstrate better performance on intelligibility of spoken words when the hearing of these words is associated with speechreading. The importance of this stimulus in strengthening the discrimination was so notorious that one may conclude that observation of the interlocutor's face works as a “third ear”.

Given the relevance of the results obtained, their publication was considered important, although the study presents limitations regarding the sample size, mainly due to the number of refusals. An improvement in the statistical significance of the results would be possible with a more appropriate sample, if a greater loss of discrimination (increase in SRT) correlates with an increasing importance of speechreading and if there is variability in the gender and/or age of the patients, despite the degree of hearing loss on presbycusis presenting variability due to genetic and environmental factors.

The results of this study are in line with others conducted in sensorineural hearing loss, but where the cause was not presbycusis. These studies found that individuals with this hearing loss had a better speechreading ability,^10^, ^11^, ^12^, ^13^, ^14^, ^15^ and disagree only with the results of one study.^16^ This finding can be explained by the routine use of this ability on hearing impairment, developed with the purpose of overcoming the hearing loss and improving communication and, consequently, improving self-esteem and sociability.^17^, ^18^ The individual's educational level also appears to be relevant to speechreading in hearing impairment.^19^

Another important issue that this study raises, in terms of the importance of speechreading in these patients, is the need to understand the role of hearing rehabilitation (including lip-reading therapy and teaching of situational and behavioral strategies) and hearing aid fitting. This rehabilitation may allow an improvement in the ability of speechreading, with a positive impact on the patient's life.^15^ It would be important to include rehabilitation before and during the prosthesis fitting, allowing for maximum use of auditory and visual information, enabling effective communication in social and family life^4^, ^20^ and preventing lack of adaptation to hearing aid devices.^21^

## Conclusions

It is concluded that the individuals of this study, whose native language is PPt and who have hearing disabilities from presbycusis, demonstrate better performance on intelligibility with speechreading. More extensive studies are needed, in particular correlating the importance of speechreading with age and the degree of hearing loss. It would also be important to broaden the understanding of communication in presbycusis and extrapolate the importance of hearing rehabilitation and hearing aid fitting in these patients, providing better social integration and better quality of life.

Study carried out at the Department of Otolaryngology of Egas Moniz Hospital, Centro Hospitalar de Lisboa Ocidental (CHLO), Lisbon, Portugal.

## Conflicts of interest

The authors declare no conflicts of interest.
